# Primary care consultations after hospitalisation for pneumonia: a large population-based cohort study

**DOI:** 10.3399/BJGP.2020.0890

**Published:** 2021-03-09

**Authors:** Vadsala Baskaran, Fiona Pearce, Rowan H Harwood, Tricia M McKeever, Wei Shen Lim

**Affiliations:** Department of Respiratory Medicine, Nottingham University Hospital NHS Trust; clinical research fellow, Faculty of Medicine and Health Sciences, University of Nottingham; National Institute for Health Research (NIHR) Nottingham Biomedical Research Centre, Nottingham.; Faculty of Medicine and Health Sciences, University of Nottingham; NIHR Nottingham Biomedical Research Centre, Nottingham.; Faculty of Medicine and Health Sciences, University of Nottingham, Nottingham.; Faculty of Medicine and Health Sciences, University of Nottingham; NIHR Nottingham Biomedical Research Centre, Nottingham.; Department of Respiratory Medicine, Nottingham University Hospital NHS Trust; honorary professor of respiratory medicine, NIHR Nottingham Biomedical Research Centre, Nottingham.

**Keywords:** general practice, pneumonia, respiratory infection

## Abstract

**Background:**

Up to 70% of patients report ongoing symptoms 4 weeks after hospitalisation for pneumonia; the impact on primary care is poorly understood.

**Aim:**

To investigate the frequency of primary care consultations after hospitalisation for pneumonia, and the reasons for consultation.

**Design and setting:**

A population-based cohort study in England using a UK primary care database of anonymised medical records (Clinical Practice Research Datalink [CPRD]) linked to Hospital Episode Statistics (HES).

**Method:**

Adults with the first International Classification of Diseases, 10th Revision (ICD-10) code for pneumonia (J12–J18) recorded in HES between July 2002 and June 2017 were included. Primary care consultation within 30 days of discharge was identified as the recording of any medical Read code (excluding administration-related codes) in CPRD. Competing-risks regression analyses were conducted to determine the predictors of consultation and antibiotic use at consultation; death and readmission were competing events. Reasons for consultation were examined.

**Results:**

Of 56 396 adults, 55.9% (*n* = 31 542) consulted primary care within 30 days of hospital discharge. The rate of consultation was highest within 7 days (4.7 per 100 person–days). The strongest predictor for consultation was a higher number of primary care consultations in the year before index admission (adjusted subhazard ratio [sHR] 8.98, 95% confidence interval [CI] = 6.42 to 12.55). The most common reason for consultation was for a respiratory disorder (40.7%, *n* = 12 840), 11.8% for pneumonia specifically. At consultation, 31.1% (*n* = 9823) received further antibiotics. Penicillins (41.6%, *n* = 5753/13 829) and macrolides (21.9%, *n* = 3029/13 829) were the most common antibiotics prescribed.

**Conclusion:**

Following hospitalisation for pneumonia, a significant proportion of patients consulted primary care within 30 days, highlighting the morbidity experienced by patients during recovery from pneumonia.

## INTRODUCTION

Community acquired pneumonia (CAP) accounts for 5%–12% of lower respiratory tract infections (LRTIs) presenting to primary care in the UK.^1^^,^^2^ Little is known about the morbidity related to recovery from pneumonia. A systematic literature review of patient reported outcomes in CAP found limited research suggesting that up to 70% of patients report at least one symptom 4-weeks post-discharge, the most common symptom being fatigue, followed by cough and dyspnoea.^3^

Readmission to hospital is common. A meta-analysis estimated the pooled 30-day readmission rate to be 10%, with 31% of readmissions due to pneumonia-related reasons.^4^ In the UK, 30-day readmission following CAP has increased from 10.5% in 2009/10 to 14.6% in 2018/19.^5^ In contrast, the impact on primary care is much less understood. In a small study of working age adults (aged <65 years, *n* = 108) discharged from hospital following admission for CAP, 59% consulted primary care within 28 days of discharge.^6^

The aim of this study was to address the gap in knowledge about the impact on primary care following hospitalisation for pneumonia, with the specific objectives of determining the rate and predictors of consultation, the reasons for primary care consultations and hospital readmissions, and antibiotic use at consultation.

## METHOD

### Data sources

The Clinical Practice Research Datalink (CPRD) is a UK government research service that provides anonymised electronic health records from general practices, with established linkages to non-primary care data such as hospitalisation data from Hospital Episode Statistics (HES) and death registration data from the Office for National Statistics (ONS). HES admitted patient care data contained details of all admissions to NHS hospitals in England from 1 April 1997 to 30 November 2018, with diagnostic data coded using International Classification of Diseases, 10th Revision (ICD-10). The ONS death registration data included all deaths registered during the coverage period of between 2 January 1998 and 14 January 2019.

### Study population and follow-up

Adults aged ≥18 years with the first episode of hospitalisation for pneumonia recorded in HES between 1 July 2002 and 30 June 2017 were included. The epidemiological year definition of July to June was used as the unit of time to avoid the winter peak of pneumonia traversing two calendar years. Pneumonia was defined as ICD-10 codes J12–J18 recorded as the primary code for the first episode of hospitalisation. Patients were excluded if they did not have data that met the minimum quality criteria for use in research, had <1 year of time registered to the practice before admission, or were admitted for at least a day in the 10 days preceding the index admission. Patients were followed up from day 1 after the date of discharge from hospital to either the first primary care consultation, end of data collection (30 days), date of transfer out of practice, date of last data collection for the practice, or date of death, whichever came first.

**Table:** How this fits in

Readmissions after hospitalisation for pneumonia occur in 10%–14% of adults, and are increasing in the UK. Up to 70% of patients report ongoing symptoms 4-weeks post-discharge. This study found that approximately 56% of adults consult primary care within 30 days after hospitalisation for pneumonia. Of those, 40.7% consult for a respiratory disorder and 31.1% receive ≥1 courses of antibiotics. To the authors’ knowledge, this is the first study to describe the previously unrecognised, but substantial, morbidity experienced by patients during recovery from pneumonia.

### Definitions

Primary care consultation was considered to have occurred if medical Read codes were recorded after the date of discharge from hospital; administration-related codes were excluded to capture face-to-face consultations.^7^^,^^8^ If multiple Read codes were recorded in a day per patient, this was counted as a single consultation. Validated codelists were used for pneumonia, smoking status, alcohol consumption, Charlson Comorbidity Index (CCI), and specific comorbidities of interest.^9^^–^^11^ In addition to the common reasons for consultations — respiratory, digestive, genitourinary, and cardiac disorders — constitutional symptoms and cognitive disorder were categorised according to Read codes. Read codes for antibiotics were categorised according to the British National Formulary (BNF) listing in Section 5.1 (antibacterial drugs), excluding anti-tuberculosis and anti-leprotic drugs.^12^

### Statistical analysis

Age was fitted as a categorical variable following the likelihood ratio test (age categories: 18–49, 50–64, 65–74, 75–84, and ≥85 years). The 2015 English Index of Multiple Deprivation (IMD) was used as a composite measure of material deprivation at the patient level.^13^ Time to consultation was measured from day 1 after discharge from hospital to the first consultation at primary care. Based on the first episode of consultation per patient, rates of consultation per 100 person–days for ≤7 days and ≤30 days were determined. Characteristics of adults who consulted were compared to those who did not consult. Predictors of consultation were determined from published literature on consultations for acute LRTI based on a postulated similarity between pneumonia and non-pneumonic acute LRTI in this regard.^14^ There were missing data in smoking status (2.9%), alcohol consumption (15.1%), and IMD score (0.1%). Multiple imputation using chained equations was performed with 10 imputed datasets for smoking status and alcohol consumption, respectively. Univariate and multivariate competing-risks regression analyses were used, with death and readmission as competing events. Univariate analyses were conducted to investigate the association between primary care consultation and each variable: age; sex; smoking status (never smoked, ex-smoker, or current smoker); alcohol consumption (non-drinker, former, occasional, moderate [≤14 units/week], or heavy drinker [>14 units/week]); length of hospital stay (≤3, 4–7, or >7 days); previous primary care consultations in the year before admission for pneumonia (<5, 5–15, or >15 consultations); IMD quintile (score of 1 [least deprived] to 5 [most deprived], or unknown); practice region (West Midlands was selected as the reference region as it was representative of England by population size, age, and sex^15^); and presence of comorbidities. Variables that were considered associated in the univariate analyses (*P*<0.05) were included in a multivariable backward logistic regression model with imputed data; age and sex were *a priori* variables. The CCI and specific comorbidities of interest were included in separate multivariate models.

The proportions for reasons of consultations were calculated for all patients who consulted, with subanalyses for those who consulted before readmission or death. The top 20 reasons for hospital readmission were determined. The number of antibiotic prescriptions, frequency of antibiotic courses (multiple antibiotics prescribed at a single consultation were counted as a single course), and the type of antibiotics prescribed at primary care consultation were examined. Univariate and multivariate logistic regression analyses were performed to investigate predictors of antibiotic prescription at consultation. Statistical analyses were performed using Stata (version 15).

## RESULTS

**Figure 1.**
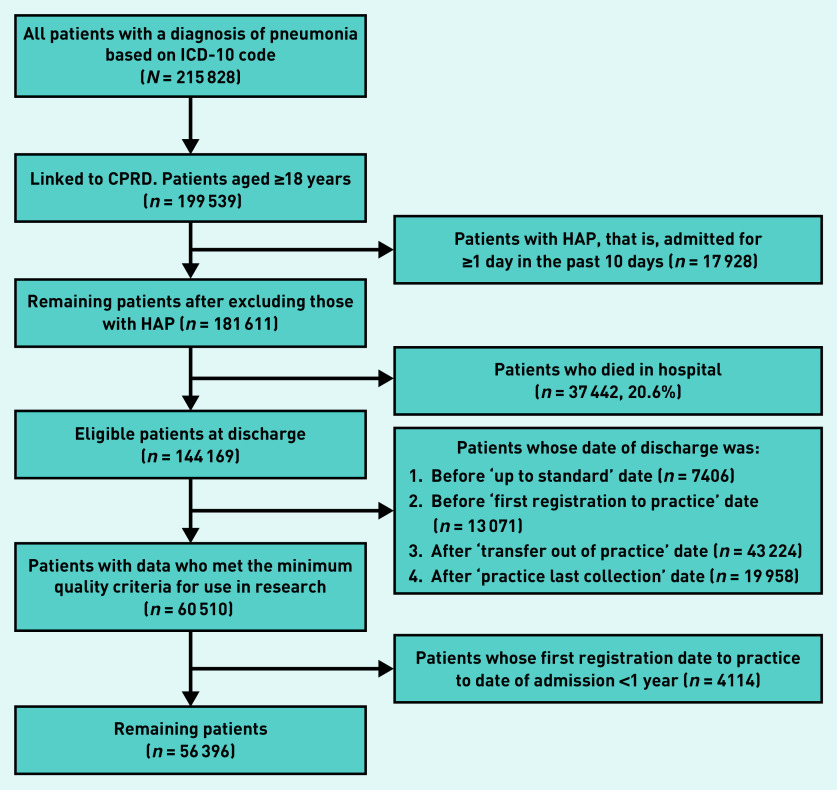
**Flowchart of study population. CPRD = Clinical Practice Research Datalink. HAP = hospital-acquired pneumonia. ICD-10 = International Classification of Diseases, 10th Revision.**

Over the 15-year study period, there were 215 828 patients admitted to hospital with ICD-10 codes for pneumonia (Figure 1), and after exclusions, the study cohort comprised 56 396 patients. Table 1 shows the characteristics of the study population. Median age of the study cohort was 75 years (interquartile range [IQR] 61–84 years) and 49.7% were male. During the 30-day followup, 16.0% (*n* = 9051) were readmitted to hospital and 6.1% (*n* = 3446) died after discharge from hospital.

Primary care consultation occurred in 27.7% (*n* = 15 626) and in 55.9% (*n* = 31 542) of patients within 7 days and 30 days of discharge, respectively. The rate of first consultation was highest within 7 days of hospital discharge at 4.7 per 100 person–days and declined to 3.3 per 100 person–days within 30 days of hospital discharge. Of those who consulted within 30 days, 47.7% (*n* = 15 056) consulted ≥2 times (data not shown).

**Table 1.** Characteristics of the overall study population

**Variable**	**Overall study population, *n* (%)**
**Patients**	56 396 (100)

**Age, years**	
18–49	8208 (14.6)
50–64	8830 (15.7)
65–74	10 499 (18.6)
75–84	15 317 (27.2)
≥85	13 542 (24.0)

**Sex**	
Male	28 002 (49.7)
Female	28 394 (50.4)

**IMD score (patient-level)**	
1 (least deprived)	10 596 (18.8)
2	11 407 (20.2)
3	11 909 (21.1)
4	11 263 (20.0)
5 (most deprived)	11 171 (19.8)
Unknown	50 (0.1)

**Practice region**	
West Midlands	6990 (12.4)
North West	9855 (17.5)
Yorkshire & The Humber	1926 (3.4)
East Midlands	1294 (2.3)
North East	1454 (2.6)
East of England	5589 (9.9)
South West	7541 (13.4)
South Central	7031 (12.5)
London	7114 (12.6)
South East Coast	7602 (13.5)

**Charlson Comorbidity**	
**Index score**	
0	13 636 (24.2)
1	12 290 (21.8)
2	9912 (17.6)
3	7777 (13.8)
4	5096 (9.0)
≥5	7685 (13.6)

IMD = Index of Multiple Deprivation.

### Factors associated with consultation

The strongest predictor of consultation was previous consultation behaviour — specifically, having consulted >15 times in the year before the index admission for pneumonia (adjusted subhazard ratio [sHR] 8.98, 95% confidence interval [CI] = 6.42 to 12.55) (Table 2). Other factors independently associated with a higher probability of consultation included: current and ex-smoking status compared to never smokers; length of hospital stay of between 4 days and 7 days compared to ≤3 days; CCI score ≥3; and co-existing chronic heart and lung conditions, as well as diabetes mellitus. Age ≥85 years was associated with a lower probability of consultation, whereas age 50–74 years was associated with a higher probability of consultation, compared to age 18–49 years. Increasing social deprivation was associated with lower probabilities of consultation. Geographical variation was observed, with the lowest probability of consultation in the South East Coast compared to the West Midlands. Sex and alcohol consumption were not independently associated with consultation.

**Table 2.** Univariate and multivariate competing-risks regression analyses investigating the predictors of primary care consultation after hospitalisation for pneumonia in the first 30 days after discharge

**Variable**		**Did not consult, *n* (%)**	**Consulted, *n* (%)**	**Univariate CRR sHR (95% CI)**	**Multivariate CRR sHR (95% CI)**		***P*-value**	
**Patients**		24 854 (44.1)	31 542 (55.9)	—	—		—	
	
**Age, years**								
18–49		4017 (48.9)	4191 (51.1)	Reference	Reference		—	
50–64		3755 (42.5)	5075 (57.5)	1.18 (1.13 to 1.23)	1.08 (1.04 to 1.13)		<0.001^a^	
65–74		4277 (40.7)	6222 (59.3)	1.24 (1.19 to 1.28)	1.08 (1.03 to 1.12)		<0.001^a^	
75–84		6459 (42.2)	8858 (57.8)	1.21 (1.16 to 1.25)	1.03 (0.99 to 1.07)		0.161	
≥85		6346 (46.9)	7196 (53.1)	1.10 (1.06 to 1.14)	0.95 (0.91 to 0.99)		0.018^a^	
	
**Sex**								
Male		12 277 (43.8)	15 725 (56.2)	Reference	Reference		—	
Female		12 577 (44.3)	15 817 (55.7)	0.99 (0.96 to 1.01)	0.99 (0.97 to 1.01)		0.32	
	
**Smoking status**								
Never		8036 (45.5)	9634 (54.5)	Reference	Reference		—	
Ex-smoker		5970 (42.3)	8149 (57.7)	1.08 (1.05 to 1.11)	1.03 (1.00 to 1.06)		0.03^a^	
Current		9897 (43.1)	13 061 (56.9)	1.06 (1.03 to 1.09)	1.03 (1.00 to 1.06)		0.027^a^	
Unknown		951 (57.7)	698 (42.3)	—	—		—	
	
**Alcohol status**								
Non-drinker		5559 (44.2)	7016 (55.8)	Reference	—		—	
Former drinker		1164 (41.1)	1667 (58.9)	1.08 (1.03 to 1.14)	—		—	
Occasional drinker		3276 (42.4)	4454 (57.6)	1.04 (1.00 to 1.08)	—		—	
Moderate drinker		7717 (42.4)	10 499 (57.6)	1.03 (1.00 to 1.06)	—		—	
Heavy drinker		2896 (44.2)	3663 (55.8)	0.98 (0.94 to 1.02)	—		—	
Unknown		4242 (50.0)	4243 (50.0)	—	—		—	
	
**CAP admission year**								
2002/03–2004/05		3296 (44.6)	4094 (55.4)	Reference	Reference		—	
2005/06–2006/07		2650 (41.3)	3764 (58.7)	1.09 (1.05 to 1.14)	1.03 (0.99 to 1.08)		0.144	
2007/08–2008/09		3387 (42.5)	4576 (57.5)	1.05 (1.01 to 1.09)	0.98 (0.94 to 1.02)		0.256	
2009/10–2010/11		4157 (43.7)	5357 (56.3)	1.03 (0.98 to 1.07)	0.95 (0.91 to 0.99)		0.008^a^	
2011/12–2012/13		4416 (43.6)	5717 (56.4)	1.04 (1.00 to 1.08)	0.95 (0.91 to 0.99)		0.014^a^	
2013/14–2014/15		3984 (44.1)	5042 (55.9)	1.04 (1.00 to 1.08)	0.95 (0.91 to 0.99)		0.015^a^	
2015/16–2016/17		2964 (49.8)	2992 (50.2)	0.88 (0.84 to 0.93)	0.82 (0.79 to 0.87)		<0.001^a^	
	
**Length of stay, days**								
≤3		7694 (44.9)	9455 (55.1)	Reference	Reference		—	
4–7		6351 (42.1)	8743 (57.9)	1.07 (1.04 to 1.10)	1.04 (1.01 to 1.07)		0.019^a^	
>7		10 809 (44.8)	13 344 (55.2)	1.03 (1.01 to 1.06)	1.00 (0.97 to 1.02)		0.852	
	
**Primary care consultations in the previous year**								
0		358 (91.1)	35 (8.9)	Reference	Reference		—	
<5		1108 (61.3)	700 (38.7)	5.31 (3.77 to 7.48)	5.11 (3.63 to 7.21)		<0.001^a^	
5–15		4039 (50.7)	3931 (49.3)	7.27 (5.20 to 10.16)	7.05 (5.04 to 9.87)		<0.001^a^	
>15		19 349 (41.9)	26 876 (58.1)	9.30 (6.66 to 12.99)	8.98 (6.42 to 12.55)		<0.001^a^	
	
**IMD score (patient level)**								
1 (least deprived)		4617 (43.6)	5979 (56.4)	Reference	Reference		—	
2		5038 (44.2)	6369 (55.8)	0.98 (0.94 to 1.01)	0.96 (0.93 to 0.99)		0.019^a^	
3		5116 (43.0)	6793 (57.0)	1.01 (0.98 to 1.04)	0.99 (0.96 to 1.03)		0.585	
4		5053 (44.9)	6210 (55.1)	0.95 (0.92 to 0.99)	0.93 (0.90 to 0.96)		<0.001^a^	
5 (most deprived)		5002 (44.8)	6169 (55.2)	0.96 (0.93 to 0.99)	0.91 (0.88 to 0.94)		<0.001^a^	
Unknown		28 (56.0)	22 (44.0)	0.73 (0.48 to 1.10)	0.78 (0.51 to 1.18)		0.237	
	
**Practice region**								
West Midlands		2878 (41.2)	4112 (58.8)	Reference	Reference		—	
North West		4160 (42.2)	5695 (57.8)	0.97 (0.93 to 1.01)	0.98 (0.94 to 1.02)		0.245	
Yorkshire & The Humber		857 (44.5)	1069 (55.5)	0.89 (0.84 to 0.95)	0.89 (0.83 to 0.95)		<0.001^a^	
East Midlands		533 (41.2)	761 (58.8)	1.00 (0.92 to 1.07)	1.01 (0.93 to 1.09)		0.846	
North East		568 (39.1)	886 (60.9)	1.05 (0.98 to 1.12)	1.05 (0.98 to 1.13)		0.154	
East of England		2246 (40.2)	3343 (59.8)	1.03 (0.99 to 1.08)	1.04 (0.99 to 1.08)		0.12	
South West		3268 (43.3)	4273 (56.7)	0.93 (0.89 to 0.97)	0.92 (0.89 to 0.96)		<0.001^a^	
South Central	3116 (44.3)		3915 (55.7)	0.91 (0.87 to 0.95)		0.90 (0.86 to 0.94)	<0.001^a^	
London	3492 (49.1)		3622 (50.9)	0.80 (0.77 to 0.84)		0.83 (0.79 to 0.86)	<0.001^a^	
South East Coast	3736 (49.1)		3866 (50.9)	0.80 (0.77 to 0.84)		0.81 (0.77 to 0.85)	<0.001^a^	

**Charlson Comorbidity**								
**Index score^b^**								
0	6590 (48.3)		7046 (51.7)	Reference		Reference	—	
1	5488 (44.7)		6802 (55.3)	1.10 (1.06 to 1.13)		0.99 (0.96 to 1.02)	0.562	
2	4387 (44.3)		5525 (55.7)	1.11 (1.07 to 1.15)		0.98 (0.95 to 1.02)	0.42	
3	3279 (42.2)		4498 (57.8)	1.18 (1.14 to 1.23)		1.04 (1.00 to 1.08)	0.047^a^	
4	2078 (40.8)		3018 (59.2)	1.23 (1.18 to 1.28)		1.08 (1.03 to 1.13)	0.001^a^	
≥5	3032 (39.5)		4653 (60.5)	1.28 (1.24 to 1.33)		1.14 (1.09 to 1.18)	<0.001^a^	

**Comorbidities**								
COPD	4666 (39.5)		7132 (60.5)	1.14 (1.12 to 1.17)		1.05 (1.01 to 1.08)	0.003^a^	
Asthma	5351 (40.1)		7996 (59.9)	1.12 (1.09 to 1.15)		1.06 (1.03 to 1.08)	<0.001^a^	
Chronic lung disease^c^	391 (43.6)		505 (56.4)	1.00 (0.92 to 1.09)		—	—	
Congestive cardiac failure	2251 (39.9)		3397 (60.1)	1.15 (1.11 to 1.19)		1.14 (1.10 to 1.18)	<0.001^a^	
Myocardial infarction	2125 (40.3)		3143 (59.7)	1.12 (1.08 to 1.17)		1.10 (1.06 to 1.15)	<0.001^a^	
Other cardiac diseases^d^	9755 (41.8)		13 571 (58.2)	1.11 (1.08 to 1.13)		1.11 (1.09 to 1.14)	<0.001^a^	
Malignancy	5231 (42.2)		7166 (57.8)	1.07 (1.04 to 1.10)		—	—	
Chronic renal disease	4624 (42.1)		6350 (57.9)	1.08 (1.06 to 1.11)		—	—	
Cerebrovascular disease	3806 (40.5)		5584 (59.5)	1.12 (1.09 to 1.15)		—	—	
Diabetes mellitus	2701 (42.4)		3676 (57.6)	1.07 (1.03 to 1.11)		1.04 (1.01 to 1.07)	0.009^a^	
Cognitive impairment	2725 (46.7)		3109 (53.3)	0.97 (0.94 to 1.01)		—	—	
Liver disease	213 (40.1)		318 (59.9)	1.09 (0.98 to 1.21)		—	—	

a *Signify a* P *-value of <0.05.*

b Charlson Comorbidity Index was added to a separate multivariate model with all the listed variables except specific comorbidities.

c Chronic lung disease excluding COPD and asthma.

d Other cardiac diseases excluding CCF and MI (for example, hypertension, arrhythmias, valvular heart disease, conduction disorder of the heart, pericarditis, and myocarditis). CCF = congestive cardiac failure. COPD = chronic obstructive pulmonary disease. CRR = competing risks regression. IMD = Index of Multiple Deprivation. MI = myocardial infarction. sHR = subdistribution hazard ratio.

### Reasons for consultations and readmissions

The most common reason for primary care consultation was for a respiratory disorder (40.7%), with 11.8% consulting for pneumonia specifically (Table 3). A small proportion of patients consulted for constitutional symptoms, such as fever, fatigue, loss of appetite, or general malaise. Reasons for consultation within 7 days of discharge were similar.

**Table 3.** Reasons for GP consultation after hospital discharge

	**All patients who consulted**		**Patients who consulted before readmission^a^**		**Patients who consulted before death^b^**	
**Reason for consultation^c^**	**≤ 7 days, *n* (%) (*n* = 15 626)**	**≤ 30 days, *n* (%) (*n* = 31 542)**	**≤ 7 days, *n* (%) (*n* = 648)**	**≤ 30 days, *n* (%) (*n* = 3459)**	**≤ 7 days, *n* (%) (*n* = 633)**	**≤ 30 days, *n* (%) (*n* = 2077)**
Respiratory (all)	6155 (39.4)	12 840 (40.7)	253 (39.0)	1350 (39.0)	158 (25.0)	741 (35.7)
Pneumonia specifically	2470 (15.8)	3730 (11.8)	71 (11.0)	293 (8.5)	87 (13.7)	312 (15.0)
Digestive	1196 (7.7)	3316 (10.5)	60 (9.3)	439 (12.7)	27 (4.3)	183 (8.8)
Cardiac	1139 (7.3)	2732 (8.7)	50 (7.7)	274 (7.9)	58 (9.2)	209 (10.1)
Genitourinary	466 (3.0)	1629 (5.2)	26 (4.0)	179 (5.2)	16 (2.5)	76 (3.7)
Cognitive	191 (1.2)	558 (1.8)	12 (1.9)	53 (1.5)	16 (2.5)	78 (3.8)
Constitutional symptoms	379 (2.4)	1240 (3.9)	16 (2.5)	162 (4.7)	15 (2.4)	84 (4.0)

a Readmission within 30 days of discharge.

b Death within 30 days of discharge.

c Only Read codes referring to acute symptoms and disorders were included, such as acute cough, acute atrial fibrillation, or worsening cognitive impairment; excluding routine reviews for chronic conditions, or routine post-discharge consultations. The same patient could fall into multiple categories for ‘Reason for consultation’.

Of patients readmitted within 30 days of discharge, 38.2% (*n* = 3459/9051) consulted primary care before readmission. These patients had similar reasons for consulting when compared to all patients. The most common reason for readmission was pneumonia; 34.6% (*n* = 1255/3625) and 26.9% (*n* = 2431/9051) within 7 days and 30 days, respectively (see Supplementary Table S1). A large proportion of patients who died within 30 days of discharge consulted primary care before death (60.3%, *n* = 2077) (Table 3). Of these, 413 (19.9%) were for reasons of palliative care or terminal illness (*n* = 230), or cancers (*n* = 183) (data not shown).

### Antibiotic prescription at consultation

Antibiotics were prescribed in 17.3% of those who consulted within 7 days of discharge compared to 31.1% of those who consulted within 30 days (Table 4). At consultations within 7 days and 30 days of discharge, antibiotics were prescribed at the same time as respiratory Read coding in 56.4% and 48.9%, respectively (data not shown). Of those who received antibiotics at consultation, 22.8% received ≥2 courses of antibiotics within 30 days of discharge. Penicillins and macrolides were the most common antibiotics prescribed (Table 4).

**Table 4.** Antibiotic prescription at consultation after hospital discharge

	**≤ 7 days, *n* (%)**	**≤ 30 days, *n* (%)**
**Frequency of antibiotic courses**		
0	12 919 (82.7)	21 719 (68.9)
1	2582 (16.5)	7587 (24.1)
≥2	125 (0.8)	2236 (7.1)
Total^a^	15 626 (100)	31 542 (100)

**Type of antibiotics**		
Penicillin	1352 (41.9)	5753 (41.6)
Macrolide	830 (25.7)	3029 (21.9)
Tetracycline	352 (10.9)	1467 (10.6)
Quinolones	220 (6.8)	875 (6.3)
Others	474 (14.7)	2705 (19.6)
Total^b^	3228 (100)	13 829 (100)

a Counted by number of people.

b Counted by number of antibiotic courses. The total for ‘Type of antibiotics’ does not match ≥1 courses of antibiotics prescribed due to difference in the way the count was performed, as specified in footnotes ^a^ and ^b^.

Factors independently associated with higher odds of antibiotic prescription in the first week after discharge were year of pneumonia hospitalisation and pre-existing chronic obstructive pulmonary disease (COPD) or asthma. Factors independently associated with lower odds of antibiotic prescription were age ≥65 years, hospital stay ≥4 days, and practice region (East of England and London) (see Supplementary Table S2).

## DISCUSSION

### Summary

The authors found that 55.9% of patients consulted primary care within 30 days of hospital discharge, the highest rate of consultation occurring within 7 days; 40.7% of consultations were for a respiratory disorder, with almost 11.8% consulting for pneumonia specifically, and 31.1% of patients consulting received further antibiotics. Previous consultation behaviour at primary care was the strongest predictor of post-discharge consultation.

### Strengths and limitations

A major strength of this study is the large, nationally representative study cohort of >56 000 patients obtained through linkage between the CPRD and HES, two large validated medical record databases.^16^ This study dataset reflects the real-world practice of pneumonia in England covering a span of 15 years. To avoid measuring non-medically relevant consultation, administration-related Read codes were judiciously excluded.

A weakness of this study is that the study data are only from England (HES is only available for England) and therefore study results may not be generalisable to the rest of the UK. Second, a large number of patients (*n* = 87 773) were excluded because their data did not meet the minimum research quality checks, or their first practice registration date to date of admission was <1 year. These patients were younger than those included (median 71 years versus 75 years, *P*<0.0001), and there was a higher proportion of females (51.5% versus 50.4%). Those discharged to a care facility outside the catchment area of their previous primary care practice would also have been excluded from the analysis. Excluded patients may have different patterns of consultation behaviour compared to the study cohort. Third, the authors relied on ICD-10 coding for the identification of patients with pneumonia. Roughly one-third of ICD-10 coded cases of pneumonia within HES lack radiographic evidence of pneumonia and would strictly be considered cases of non-pneumonic LRTI.^5^ The vast majority of these patients are nevertheless treated clinically as having pneumonia, and inclusion of these patients in the analysis reflects routine practice. Fourth, although considerable efforts were made to ensure data quality, the authors cannot fully exclude the possibility of information bias from miscategorisation of the study exposure, confounders, and outcomes.

### Comparison with existing literature

To the authors’ knowledge, very few studies have examined the impact on primary care following hospital discharge after pneumonia. A Dutch study using electronic health records observed that only 8% of adults consulted primary care within 30 days after discharge.^17^ Their study comprised patients who were younger — the mean age range per year from 2002–2009 was 57 years (standard deviation [SD] 27.9) to 61 years (SD 24.8) — and with lower severity; combined mortality (in-hospital and within 30 days of discharge) was 7%, compared to 26.7% in the current study. Two Spanish studies (a prospective cohort study at a tertiary hospital, *n* = 934, and a multicentre clinical trial, *n* = 207) observed consultation proportions of 18%–20%.^18^^,^^19^ A three-centre UK study by Daniel *et al* of adults aged <65 years (*n* = 108) found primary care consultation occurred in 59% *.*^6^

The present study found a lower proportion of consultation due to respiratory symptoms compared to Daniel *et al* and Adamuz *et al* (69% and 75%, respectively). Direct comparison between these studies is not possible due to the use of different methodologies for measuring and categorising reasons for consultation.^6^^,^^19^ Antibiotic use at consultation in the current study (30.8%) was similar to that reported by Daniel *et al* (34.4%).^6^

Other studies have investigated the burden of reconsultations after primary care management of patients with LRTIs or acute bronchitis.^14^^,^^20^^–^^22^ The patient cohorts in these studies mostly involve adults with self-limiting LRTIs in whom the challenge is the avoidance of overuse of antibiotics and managing patient expectation. In these patient groups, reconsultations were observed in 20%–33%. Similar to other research in LRTI consultations not requiring hospital admission, the current study found that a previous history of consultation was a strong predictor of post-discharge consultation.^14^^,^^23^ The provision of patient information leaflets or delayed prescriptions in the management of LRTIs has been shown to reduce reconsultation.^23^^–^^25^ Such strategies may be relevant in managing patients on discharge from hospital. In addition, existing integrated post-discharge care pathways between primary and secondary care may provide applicable approaches to improving the quality of care and patients’ experiences following pneumonia.^26^^,^^27^

### Implications for research and practice

After discharge from hospital, patients often continue to report persistence of symptoms, including fatigue, cough, and dyspnoea associated with functional impairment for several weeks.^3^ Primary care consultation following hospitalisation for pneumonia may serve as a means of safety-netting, providing an opportunity for clinicians to identify deteriorating patients who need further medical intervention, or sometimes readmission. On the other hand, qualitative studies reveal that at the time of hospital discharge, patients often lack a clear understanding about the short- and long-term consequences of CAP, or the natural course of their symptoms.^28^^,^^29^ Many patients describe a sense of isolation when their experiences of relatively slow recovery do not match the expectations of relatives, carers, and even physicians.^28^^,^^29^ Ongoing unaddressed patient needs may contribute towards the high level of primary care consultation observed as patients seek reassurance of adequate recovery. Such consultations may be avoidable.

At a strategic level, lack of recognition by the public, clinicians, and policy makers of the morbidity experienced by patients during recovery from pneumonia has thus far meant that evidence-based interventions to meet patients’ needs have not been adequately developed. The health economic costs of primary care consultations are considerable. Annually, >100 000 patients are admitted to hospital in England with CAP.^30^ Assuming a cost of 30 GBP for each primary care consultation, the authors estimate post-pneumonia consultations alone to cost the NHS approximately 2 million GBP a year.^31^ These figures do not take into account any additional NHS and ecological costs from antibiotic prescribing, nor the impacts from ‘long COVID’ consequent from SARS-CoV-2 infection.^32^^–^^35^

The observation that previous consultation behaviour is strongly associated with post-pneumonia discharge consultation raises the question of whether the index pneumonia admission is a precipitating event leading to further health consequences, or whether it is only a marker of ongoing health needs. It is likely that both these explanations play some part. Further studies are required to better understand the relative contributions of these factors and to inform where to direct health improvement efforts.

A significant trend toward lower levels of post-discharge consultations was observed over the 15 years of the study (2002–2017). Over that period, concerted efforts were made nationally to improve the care of patients with CAP, including major updates of national CAP guidelines (from the British Thoracic Society in 2009 and the National Institute for Health and Care Excellence in 2014).^1^^,^^2^ A corresponding decrease in mortality from CAP over a 10-year period (2009/10–2018/19) was also observed.^5^ These initiatives may have contributed to the observed decrease in post-discharge consultations and support continued efforts in this direction through the focus on pneumonia within the *NHS Long Term Plan*.^26^
